# Opportunities and challenges on hospital preparedness to handle motorcycle accidents in Busia County, Kenya: an exploratory qualitative study

**DOI:** 10.11604/pamj.2024.47.101.40829

**Published:** 2024-03-04

**Authors:** Olipher Makwaga, Ferdinard Adungo, Tom Mokaya, Elizabeth Echoka, Matilu Mwau

**Affiliations:** 1 Kenya Medical Research Institute, Busia, Kenya

**Keywords:** Hospitals, challenges, opportunities, motorcycle accidents

## Abstract

**Introduction:**

motorcycles continue to be a popular mode of transport in Kenya. However, the related injuries cause significant morbidity and mortality and remain to be a major and neglected public health issue. This raised the crucial need for hospital preparedness in managing morbidities and in reducing mortalities. This formed the basis of this paper which aims to document the challenges and opportunities in the healthcare system in handling motorcycle accidents in a Kenyan border town in Busia County.

**Methods:**

we drew data from an exploratory qualitative study that was carried out in 2021. All six referral hospitals purposively included in the study. The study targeted a total of 25 top level facility managers as key informants on the facility level opportunities and challenges in handling motorcycle accidents. Descriptive data were analyzed using SPSS version 20.

**Results:**

the hospitals were not well prepared to handle motorcycle accidents. The major challenges were understaffing in critical care services; inadequate/lack of equipment to handle motorcycle injuries; inadequate/lack of infrastructure i.e. surgical wards, emergency rooms, inadequate space, functional theatre; lack/inadequate supplies; overstretched referral services arising from the hinge burden of motorcycle accidents in the area; inadequate specialized personnel to provide trauma/care services; mishandling of cases at the site of accident; inability of victims to pay related bills; inappropriate identification of victims at the facility; lack/inadequate on-job training. Some opportunities that currently exist include health system interventions which are not limited to employment of more professionals, improvement of infrastructure, provision of equipment and increase of budgetary allocation.

**Conclusion:**

the study reveals vast challenges that are faced by hospitals in managing patients. This calls for the government to step in and capitalize on the proposed opportunities by the health managers to be able to manage morbidities and bring down mortalities due to motorcycle accidents.

## Introduction

Globally, it is estimated that 1.2 million people are killed in road accidents each year and as many as 50 million are injured [1,2]. Among other types of road traffic accidents, motorcycle accidents form the fatal category of road accidents [3] with reported prevalence of injuries ranging from as high as 62% in Vietnam, 22.8% in China, 12.8 - 60% in different studies in Nigeria and about 39.4% in some studies in Kenya [4-6]. Given the fact that motorcycles offer virtually no protection to the users, the risk of dying from a motorcycle accident is estimated to be 20 times higher than from a motor vehicle accident [3,7]. In developing countries such as Kenya, the growth in economy and urbanization has led to an increase in motorized two-wheeled or three-wheeled vehicles. This is one of the least safe forms of travel that has resulted in a significant increase in the proportion of road traffic injuries (RTIs) emanating from motorcycle accidents. This is a major but neglected emerging public health problem and a leading killer of the most economically productive age group (15-25 years) affecting mainly the motorcyclists, pillion passengers and pedestrians. Currently, many developing countries are already facing a huge problem of a rapidly increasing fatality and disabilities due to motorcycle-related injuries with the vulnerable groups bearing the brunt of it [7-10]. The vulnerability of motorcycle riders on the road represents the highest public health burden when expressed in disability-adjusted life years lost. Earlier studies in the United States, Rwanda, Brazil, Taiwan and Jamaica testified that public health systems are not sufficiently equipped to encounter the supplementary trauma care requirements and face major challenges in coordinated emergency responses [11]. Because public health resources have been prioritized for health system development for communicable diseases [12]. Managing and treating road traffic related injuries and trauma in Kenya is very expensive and possesses a significant challenge both to the health system and the patients [13-15].

The World Health Organization (WHO) has identified six components that define a health system [16]. These include service delivery, trained and motivated health workforce, health information systems, and health financing mechanisms, availability of essential medicines and diagnostic technologies and appropriate governance structures [16]. Health systems in all counties in Kenya are currently weak, underfunded and ill prepared to respond to emergency conditions such as RTIs. The increasing burden of motorcycle related injuries has led to increased demand for trauma care services which are often limited at the county level. There is no reliable information on the level of health system preparedness to manage motorcycle-related injuries and other road traffic accidents not only in Busia County but also in the entire country. For these reasons the study was designed to determine hospital preparedness to offer quality trauma care services in Busia County. The research question was how can hospitals in Busia County be improved to better manage motorcycle accident patients? The objective was to determine the possible measures needed to improve services offered to motorcycle accident patients in Busia County referral hospitals.

## Methods

**Study design:** the study was explorative qualitative study with key informant interviews (KIIs). Managers from Six hospitals purposely responded to interviews. Information on the availability of trauma care services and facilities for managing patients involved in motorcycle accidents was collected. Information regarding human resources, access to trauma care services, and availability of essential equipment and supplies was also collected. The KIIs were requested to respond to proposed appropriate strategies for improving and scaling up of services. For the purposes of this study, service availability referred to the physical presence of the delivery of services, health infrastructure, and core staff/trained staff, policies, equipment, medicines and commodities/supplies.

### Study setting and site

The study was conducted in Busia County, Kenya from March to December 2021. The county has one level five referral hospital and five level four referral hospitals. The current study involved all of these referral hospitals. The key informant interviews were conducted in the following six referral hospitals: Teso, Alupe, Khunyangu, Sio Port, Port Victoria sub-counties hospitals and Busia County referral hospital. Busia County is located at the borders of Kenya-Uganda and the West of Uganda. It is also located at the South of Lake Victoria and Siaya County. It borders Bungoma and Kakamega counties towards North and East respectively. The county is characterized by cross border movement of Kenyan and Ugandan pedestrians and motorcycle riders with an aim of trading. The county has a population size of 893,681 (Kenya National Bureau of Statistics, 2019 [17]. The county has approximately 15,000 motorcycle riders [18].

**Study participants:** all the six referral hospitals in Busia County participated in the KII relating to services offered to trauma and motorcycle accident patients. For each referral hospital, only top health managers were interviewed. The managers were representatives of the facility and were appointed by the hospital top management to take part in the study.

**Variables:** hospitals, gender, staffing, equipments, infrastructure, supplies, specialized personnel, inadequate referral systems, mishandling of cases among others.

**Sample size determination:** for the KII, purposive sampling was used to select respondents to ensure that possible ranges of experiences are represented. There is no formulae for calculating sample size for qualitative study, however, Mira Crouch and Heather McKenzie recommend a sample size of 20 for quality interview based research [19]. The number of KII interviewed in this study were 25 of which four to five responded were drawn from each hospital.

**Bias:** biasness in the study was addressed in a manner that all the core departments offering trauma and care services were represented. At least 4 top managers were requested to respond to the same questions, these were Departmental heads of nurses, Administrators, Clinical Officers, Medical Doctors, Physiotherapists and Orthopedic.

**Data management and analysis plan:** data collected using an interview KII guide focusing on the broad themes paper-based questionnaires was double entered (independently) into computer databases within two days of collection. Hard copies were scanned for back up storage and the questionnaires were stored in lockable and secured cabinets. All data and information in the computers was password protected, verified before analysis. Descriptive statistics in SPSS version 20 was used to compute frequencies of variables.

### Ethical concerns: Institutional Review Board Approval

Scientific and ethical clearance was obtained from KEMRI Scientific and Ethics Review Unit (SERU). This study was approved by the ethical review unit of Kenya Medical Research Institute number study KEMRI/SERU/CIPDCR/008/3639. Permission to conduct situational analysis of the hospitals was sought from the hospital managers. Informed consent was sought from the participants.

## Results

In 2021, all the six health referral hospitals in Busia County, Kenya were considered for this study. In general, a total of 25 (male 14; female 11) top managers of these facilities were interviewed on the preparedness of the hospitals to handle victims who are involved in motorcycle accidents. Professionally, four Administrators, six Clinical Officers, four medical doctors, six nurses, two orthopedic personnel and three physiotherapists across the six facilities were interviewed (Table 1).

**Table 1:** socio-demographic of the participated health workers

		Alupe sub-county referral hospital	Busia County referral hospital	Khunyangu sub-county referral hospital	Kocholia sub-county referral hospital	Port Victoria sub-county referral hospital	Sio-Port-Victoria sub-county referral hospital
Gender, n=25	Male	2	2	2	3	3	2
	Female	2	2	2	2	1	2
Health professionals, n=25	Administrator	1	1	1	1	0	0
	Clinical Officer	1	1	1	1	1	1
	Medical Doctors	1	0	0	1	1	1
	Nurse	0	1	1	1	1	2
	Orthopedic	0	0	1	0	1	0
	Physiotherapist	1	1	0	1	0	0

All the managers responded that the hospitals are not well equipped and have many challenges in the handling of such patients. The challenges include general understaffing; Inadequate or lack of equipment; Inadequate or lack of infrastructure i.e. surgical wards, emergency rooms, inadequate space, functional theatre; Inadequate supplies; Inadequate ambulance services; Inadequate specialized personnel to provide trauma and care services. Other challenges: mishandling of cases at the site, patients are unable to pay bills; unable to identify patients both at the site of accident and at the facility; Inadequate on-job training (OJT), Some of the proposed opportunities by the managers for improvement included establishment of patient´s accidents handling policy, establishment of emergency department, provision of trauma care/management training, upgrading of infrastructure, provision of essential equipment, employing of more professionals, timely referrals, increase of budgetary allocation, avail emergency teams to collect the victims, improve the salaries of the health workers to keep them at one place, confirm staffs on contract into permanent jobs, avail health insurance policy to all the riders (Table 2).

**Table 2:** challenges and proposed solutions on how to handle motorcycle victims involved in accident

Responses on why the hospitals are not prepared to handle victims involved in motorcycle accidents, n=25							
**Challenges**	General understaffing,	Inadequate or lack of equipment such as X-ray, CT scan, Ultrasound, MRI, wheel chairs,	Inadequate or lack of infrastructure e.g. surgical wards, emergency rooms, inadequate space, functional theatre, orthopedic	Inadequate supplies e.g. fuel for ambulances/backup generators drugs, blood for transfusion,	Inadequate specialized personnel to provide trauma and care services, orthopedic doctors, physiotherapy/orthopedic.	Inadequate referral services	Mishandling of cases at the site
**Frequency of responses**	Yes=19 No=6	Yes=22 No=3	Yes=21 No=4	Yes=15 No=10	Yes=20 No=5	Yes=13 No=12	Yes=10 No=15
**Challenges**	inability of patients to pay bills	Inability to identify patients at the site and at the facility	Inadequate on-job training (OJT)	Inadequate policies on how to handle accident victims	Poor remuneration of health workers		
**Frequency of Responses**	Yes=13 No=12	Yes=13 No=12	Yes=13 No=12	Yes=10 No=15	Yes=8 No =17		
**Responses to proposed solutions in handling of victims involved in motorcycle accidents, N=25**							
**Proposed solutions**	Employ more and professionals’ health workers	Trauma care/management training	Separate emergency department	Tutorials	Upgrade infrastructure i.e. emergency rooms	Provision of essential equipment and supplies/drugs	All riders to have NHIF, 10;
**Frequency of Responses**	Yes=16 No=9	Yes=23 No=2	Yes=25 No=0	Yes=22 No=3	Yes=25 No =0	Yes=24 No=1	Yes=16 No=9
**Proposed solutions**	Train riders on how to handle victims	Establish accident patient policy	Timely referrals	Financial support to hospitals	Availability of emergency team to collect the victim	Improve the salaries of health workers to keep them at one place	Confirm staffs on contract to permanent positions
**Frequency of Responses**	Yes=10 No=15	Yes=9 No=16	Yes=9 No=16	Yes=9 No=16	Yes=11 No=14	Yes=12 No=13	Yes=15 No=10

## Discussion

Healthcare services in Africa are generally in impracticable circumstances with poor healthcare results. In the current study, slightly more males than female health workers were interviewed confirming earlier study [20]. This is because the female health workforce remains under represented in health leadership positions in Africa [21]. Past studies have reported challenges facing hospitals in Africa, and our chief outcomes were to a large scope comparable to previous studies [22-24]. The leading major five challenges to health care services in the current study include Inadequate/lack of equipment, Inadequate/lack of infrastructure, inadequate specialized personnel, General understaffing, and inadequate supplies. As much as availability of proper and functional equipment contributes to the efficiency of healthcare services [25], Inadequate/lack of functional equipment in the sampled facilities in the current study was found to be the topmost hindrance to provision of standard health care to those involved in motorcycle accidents. Among the equipment, the most functional primary machine like X-ray was available in only one referral hospital (Busia) in the whole of Busia County. In fact the machine is operational during the day and not at night due to understaffing. Machines like computed tomography (CT) scan, Ultrasound, and Magnetic Resonance Imaging (MRI) are available in the same facility, however, they are not operational due to frequent stock-outs and understaffing. The machines that exist within the Orthopedic and physiotherapist department in these facilities are obsolete. This scenario is similar with the study conducted in the previous study in Kenya in that lack of medical equipment hinders provision of Universal health care in Kisumu County [26]. Similarly, the current study reports alike outcome with previous studies conducted in other African countries [27].

Health professionals need good infrastructure to be able to provide standard healthcare services. However, Inadequate/lack of infrastructure and poor quality infrastructures like minor theatres, emergency rooms and other vital rooms in these facilities make it hard for the health professionals to work effectively. Frequent fluctuation of electricity in most of these facilities make it hard for health workers to offer theatre services on time. In fact those who are involved in accidents wait for longer periods due to electricity shortage. The back-up generators exist in most of the hospitals, however the fuel is not always available hindering service provision by the health workers. Understaffing in the studied hospitals was another challenge in the provision of effective healthcare services. Lack/inadequate of essential health professionals like orthopedic and physiotherapists in some of these facilities make it hard for those involved in accidents to receive standard healthcare services. The outcomes of the understaffing and inadequate specialized personnel in the current study conform with the previous studies conducted in other African countries which reported that these challenges are the top hindrances to health care provision in Africa [28,29]. However, the percentage recorded for these challenges in the current study is higher compared to the previous. The reasons attributable to this could be due to higher sample size (77) in the previous studies and also inclusion of numerous study countries unlike the current study which considered 25 responders from one county and country. Continuous and adequate provision of routinely utilized supplies is a vital element in managing the diseases and other health conditions [29]. However, inadequate supplies which include lack and/or shortage of medicines in the studied hospitals hamper healthcare service provision and especially to those involved in accidents. Inadequate of other daily used supplies make it hard for the health workers to provide optimal services. This scenario was similarly reported in other studies elsewhere [30] which reported shortages of medicines in a hospital due to procurement challenges. However our study did not find out the reasons for shortage of medicines and other routinely utilized supplies.

Our study shed light on other challenges hindering the provision of trauma healthcare services in Busia hospitals. These include inadequate referral services, mishandling of cases at the site of accident, inability of patients to pay bills, inability to identify victims of accidents at the facility, inadequate on-job training (OJT) of health workers. These challenges complicate provision of credible trauma and healthcare services in these facilities. Operational referral systems from one hospital to a higher one is vital to save lives and ensure worth and a scale of care [31]. The efficiency of referral systems in Busia hospitals depends on the availability of both the ambulances and also the fuel. Unfortunately, the whole of Busia County has only eight ambulances and most of the time, fuel is not available to deliver those involved in accidents to other hospitals on time for further management. Generally, most motorcycle riders are low income earners and when they are involved in an accident, they are unable to meet the high cost of medical services offered to them. In fact, they are detained over unpaid bills till the family member resorts to other measures of paying like ‘harambee’ or selling of land. For those who don't have such measures to enable them to pay, they are released after some day´s detention and this results in income loss to the facilities. As much as identification of patients at the hospital is associated with patient care, safety, payment and data sharing [32]. The health managers in the studied hospitals reported that, they sometimes fail to identify the victims of motorcycle accidents and this most of the time complicates hospital bill payment and data sharing. In most cases, OJT has an impact on Institutional productivity despite of other challenges facing hospitals, this is because OJT improves the quality of the human resource of an institution [33], however, interviewed managers in the studied hospitals described that, inadequate OJT exists in the hospitals and this kills their moral to offer effective services to the patients. Another challenge that was reported by managers was mismanaging of the patient at the site of accidents by other motorcycle riders who tend to provide assistance in transporting the victim to the hospitals. This makes the victim more injured than before just after the accident.

Some of the proposed opportunities by the interviewed health workers for improvement were not different from other studies conducted in the past [25,28,31]. These included, employment of more and professional health workers, improving infrastructure such as build emergency rooms. Increase of financial support to facilities, avail emergency team to collect the victim, improvement of health workers´ salaries to keep them at one place, confirmation of staff on contracts to permanent positions and avail fuel for ambulances for adequate and timely referral services. Promotion of OJT amongst health workers and especially Trauma care/management training. The three new and current proposed solution to challenges facing the management of victims of accidents included introduction of first aid training to all the riders so as to reduce traumatizing of the victims at the site of accidents, strengthening of the universal health care by ensuring all the riders have health insurance policy especially the *National Health Insurance Fund* (NHIF) and ensuring that all the riders carry identification card while they are on duty were additional solutions and opportunities that they can be capitalized in the management of victims of motorcycle accidents.

## Conclusion

Multiple challenges accessed in this study pose a threat in the provision of essential healthcare services in the hospitals and especially to those involved in accidents. Strengthening the hospitals is important not only to employ more health professionals, general improvement of infrastructure, improve general supply chains, referral systems, clinical capacity at public hospitals, budgetary allocations and improve salaries of staffs, but also confirming those on contracts positions to permanent positions and provide insurance health cover to riders to be able to reduce mortalities due to motorcycle accidents. First aid training provision to riders would reduce trauma and more injuries to victims of accidents. Limitation of the study: our study concentrated on the collection of information from the top managers. This would underrate the general data. This is because some hidden information would have been revealed by the junior officers. We recommend in such situations, all the information should be collected from both the junior and senior health workers. Overall interpretation of the results: hospitals in Busia County are facing many challenges in the management of patients who are involved in accident. However, if these challenges can be improved, the patients can be managed well.

### What is known about this topic

- *Hospitals are facing challenges in handling patients;*
- *Regardless of Universal health care services advocated, many countries are yet to attain it to 100%;*
- *Challenges facing hospitals contribute to mortalities of victims involved in accident*.

### What this study adds

- *Proof collected in this study add-ons knowledge to the existing literature on challenges facing hospitals to handle victims involved in accidents;*
- *Shed light on the opportunities the government can capitalize on to be able to improve the management of patients involved in accidents*.
